# Combining Natural History Collections with Fisher Knowledge for Community-Based Conservation in Fiji

**DOI:** 10.1371/journal.pone.0098036

**Published:** 2014-05-21

**Authors:** Abigail S. Golden, Waisea Naisilsisili, Isikele Ligairi, Joshua A. Drew

**Affiliations:** 1 Dept. of Ecology, Evolution and Environmental Biology, Columbia University, New York, New York, United States of America; 2 Wildlife Conservation Society Fiji, Suva, Fiji; 3 University of the South Pacific, Suva, Fiji; Seagrass Ecosystem Research Group, Swansea University, United Kingdom

## Abstract

Harnessing the traditional ecological knowledge (TEK) of local communities has the potential to enhance conservation planning in developing regions. Marine protected areas (MPAs) that incorporate traditional beliefs about reef tenure are generally more successful in reaching conservation goals and ensuring the participation of local fishermen on vulnerable tropical reef systems. Fiji possesses a unique system of traditional reef management in which local clans or villages, called *mataqali*, control individual units of a reef, known as *qoliqoli*, and make independent management decisions based on traditional beliefs and conservation concerns. This is an example of a system, known as customary marine tenure, which has attracted interest from conservation scientists hoping to set up MPAs in vulnerable regions. As one example of this grassroots participation, Nagigi village on the Fijian island of Vanua Levu has expressed interest in setting up an MPA in part of its *qoliqoli* because of concerns about overfishing. In response to this interest, we took a two-pronged approach to assessing Nagigi's fishery status and conservation needs, first conducting a fishery-independent species survey using destructive sampling and then focusing on fisheries targets identified through fisher interviews. These interviews allowed us to identify heavily targeted species, assess villagers' understanding of reef dynamics over 30 or 40 years of fisheries expansion, and evaluate village support and expectations for a proposed conservation program. Based on our findings we recommend a temporary closure to be in effect for at least three years, allowing one of the more important fishery targets, *Lethrinus harak* (Forsskål, 1775; Lethrinidae), to complete at least one generation within the reserve. The methodology of matching the proposed marine protected area with the life histories and ecologies of heavily targeted species identified through fisherman and -woman interviews can offer a template for future conservation projects that seek to synthesize indigenous peoples' needs and knowledge with ecological data.

## Introduction

The last thirty years have seen a surge in interest from ecologists and conservation scientists in trying to couple formal management strategies with local indigenous communities' strategies for exploiting and managing their natural resources. These combined strategies, collectively known as community-based management, include techniques such as the temporal restriction of wildlife harvests, protection of vulnerable life-history stages of an exploited species, and resource rotation [1]. Decisions concerning when or where resource exploitation is prohibited and what resources are to be utilized at any given time are traditionally made by local leaders recognized by their community for their high status and/or harvesting prowess [2], [3]. These traditional management techniques vary across indigenous communities and between habitat types.

Many South Pacific peoples are highly dependent on marine products as a source of animal protein [4]–[6]. As a result, the health and food resources of these people are intertwined with the health of their coastal ecosystems, and communities have developed traditional management techniques to regulate their use. The most widespread form of community-based marine management in the region is the reef and lagoon tenure system, also known as customary marine tenure [5]–[8]. To manage their crucial marine resources, many cultures developed tenure systems in which a chief, clan, or family controlled a particular area of coast and regulated its exploitation for successive generations. Consequently, it was in the best interests of a particular tenure-holder to harvest from the reef in moderation, ensuring consistently high yields and avoiding social censure for overharvesting. In this way, Pacific Islander communities could prevent the situation described by Hardin [9] as the “tragedy of the commons,” in which natural resources are progressively overutilized and degraded because no single individual bears as much of the cost of overharvesting as he or she reaps of its benefits. In Fiji, reef tenure takes the form of coastal tenure areas called *qoliqoli* that are legally controlled by individual patrilineal clans known as *mataqali* [8], [10]. More recently, as development brought greater mobility, clans dispersed, and this control moved to individual villages, which could make the communal decision to temporarily ban fishing on portions of a *qoliqoli* in response to overfishing and other causes, thus helping to maintain a healthy coastal ecosystem over many generations.

One corollary of community-based management in indigenous cultures is the existence of bodies of traditional ecological knowledge (TEK) concerning the plants, animals, and environmental conditions these societies need to survive. TEK has been defined in various ways, but it is generally understood to consist of a body of knowledge passed down through generations about the relations of humans with other living beings and their environment [1], [11]. Facets of a culture's TEK include the local names for common species, knowledge about timing and location of biologically significant events such as migrations, and the ways in which local people use, perceive, and manage their natural resources [12]. Since the 1970s, ecologists have increasingly begun to consider these aspects of indigenous cultures' TEK a significant resource for making informed decisions about the management and conservation of poorly studied ecosystems, such as Pacific coral reefs and sea grass meadows [5].

Despite the importance of TEK and customary marine tenure to indigenous communities, colonization by Western powers brought economic and cultural changes that led to degradation of traditional knowledge and management systems throughout the Pacific. According to Johannes [5], three main factors contributed to the decline of marine tenure systems in Pacific Island nations under colonial rule: (a) the introduction of a market economy; (b) the devaluation of traditional authorities; and (c) new management laws imposed by colonial powers. In Fiji, these processes began to put heavy fishing pressure on the country's marine resources following World War II, with concerted fisheries development coming to the fore in the 1950s [10], [13]. International and Fijian national interests began to push for the increased commercialization of Fiji's fisheries and the shift to an export market, with the Fiji Development Bank encouraging the commercial development of even the country's remotest villages [8], [10]. This transformation led to significant overexploitation of both traditional inshore fisheries and the international offshore tuna fishery, which according to many experts may be close to collapse [10], [14]. In addition, cultural values have shifted such that some younger Fijians place less value on traditional management systems and see the ocean primarily through a commercial, rather than a traditional, lens [10]. Today, of Fiji's 410 *qoliqoli*, 70 are considered overexploited, with a further 250 fully developed at maximum sustainable yield capacity [15].

The increasing replacement of traditional fishing methods with new technologies, such as motorboats, nylon nets, spearguns, diving gear, and flashlights for night fishing, has also contributed to overharvesting in Pacific subsistence communities [16]–[18]. In places where traditional values are largely intact, such as the remote Fijian island of Ono-i-Lau, 400 kilometers from the country's capital, these new technologies have increased fishing efficiency but not overall catch [16]. In such places, subsistence still governs fishing effort and fishermen catch no more than they need for their own use [5], [16]. However, in more central locations where a cash economy is in place, fishermen use these new technologies to overfish their local reefs and seagrass habitats, selling their surplus for cash, thereby eventually increasing the fishing effort necessary to make a living from the reef.

More recently, Fiji and other Pacific countries developed marine conservation and management programs, often in the form of marine protected areas (MPAs), in response to increased concerns about overexploitation and habitat degradation. In many Western countries, such fishing recommendations would be based on hard data detailing the ecological dynamics of the fishery, and regulation would be top-down and enforced by national legislation [19]. However, Pacific Island marine ecosystems offer unique challenges to such a Westernized approach to fisheries management, due to their greater biodiversity, remoteness, and interconnectivity [19]. For instance, such coastal areas are characterized by complex interactions between coral reefs and the mangrove forest and seagrass meadow habitats, which provide nursery and foraging areas for valuable reef fish [20]. To circumvent the challenges posed by this Western model of top-down management, researchers have begun to suggest the creation of networks of small, locally protected areas based on reef tenure divisions and incorporating traditional management systems [17], [18]. In Fiji, these have taken the form of locally managed marine areas constructed around the country's existing *qoliqoli* system [3], [8], [10], [15]. Such management programs attempt to blend modern scientific techniques with the input of local stakeholders and possessors of traditional ecological knowledge.

**Figure 1:**
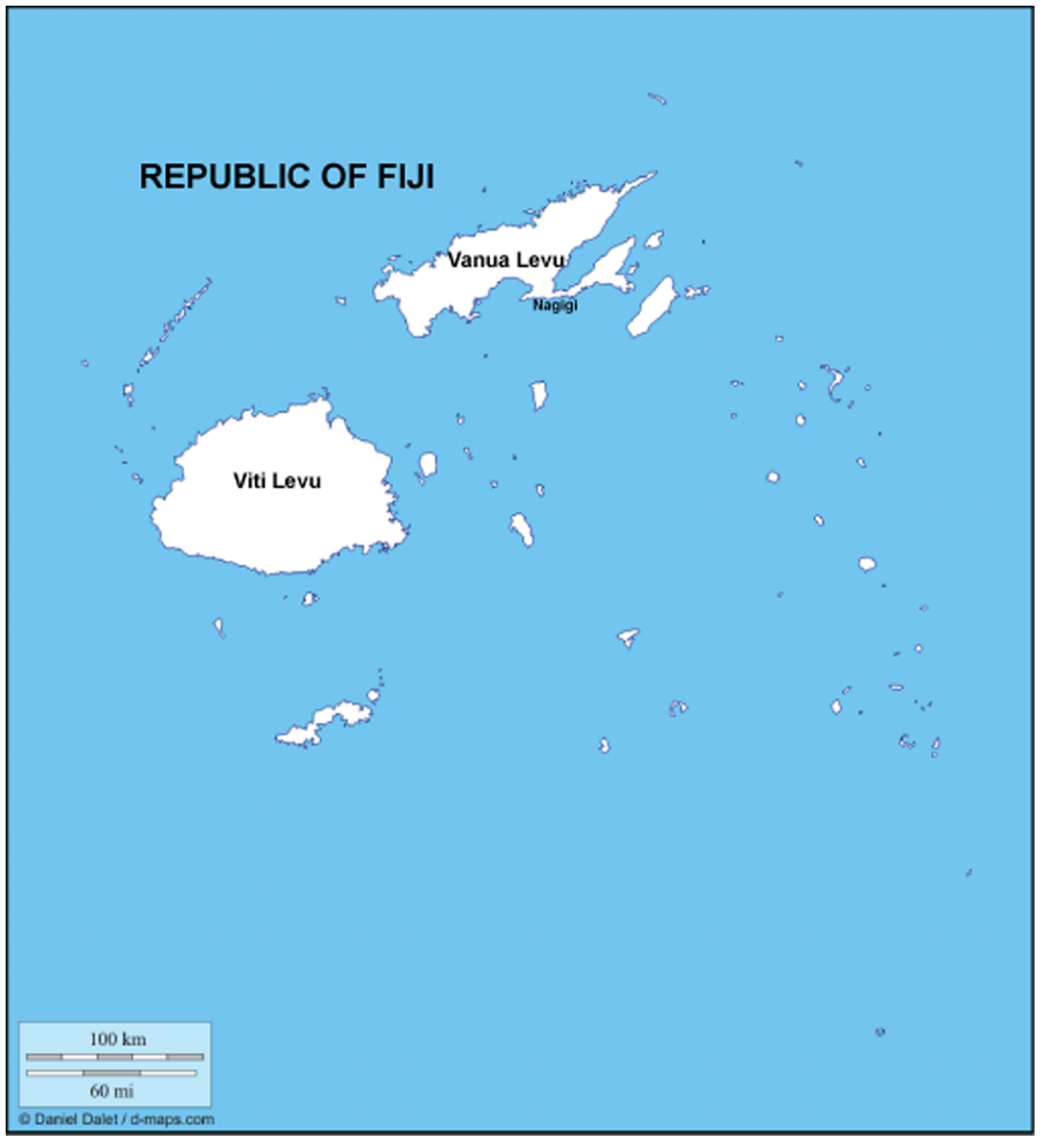
Map of Fiji used with permission of Daniel Dalet, d-maps.com.

Nagigi Village, a small fishing community on the southern coast of Vanua Levu, Fiji's second-largest island, provides one such example of community-driven demand for marine protection (Figure 1). In recent years, village leaders have expressed interest in establishing a short-term marine protected area on part of Nagigi's *qoliqoli*. As one villager put it, “For the sake of future generations, if we want to have an abundance of resources again, we should encourage an MPA on the fishing grounds. Our main concern is that if we're not aware of what's done, future generations won't know what those species are or recognize the need to gain back what they've lost” (Interview 2). Our research combined destructive sampling of reef fish to create a survey of Nagigi's biodiversity with villager interviews in an attempt to answer the following questions related to Nagigi's subsistence and artisanal fishery:

- What methods characterize subsistence and artisanal fishing in a traditionally managed Pacific Islander society, and what are the benefits that people gain from these crafts?
- What species are heavily targeted in such a society, and therefore at risk for overfishing and population collapse?
- How do villagers perceive that their reef environment has changed throughout their lives, and what do they understand to have caused these changes?
- How do villagers conceptualize marine resource management, and what conservation measures might they support to halt and reverse any negative impacts they have observed?

To answer these questions we carried out a two-part investigation into the fisheries of Nagigi. The first component was a fishery independent survey of potential target species captured in and around the Nagigi *qoliqoli*. We then used this baseline data as a launching pad for a series of interviews with Nagigi fishers to understand the extent of the fishery, to explore any differences between genders in fishing and finally to assess fishers' attitudes towards the establishment of a no-take MPA in their waters.

## Methods

### Ethics Statement

All fish were collected under the auspices of the Columbia University Animal Care Board permit to Joshua Drew (AC-AAAF6300). Every effort was made to minimize the number of samples collected, the environmental damage caused by collection, and the suffering of the animals sacrificed. For our human subject data, prior to each interview we explained the potential, albeit minimal, risks to the interviewee as well as why and how the information would be collected and personal data secured. After explaining this in both English and Fijian we obtained a signed written consent form and the interview proceeded. These procedures were reviewed and approved by the Columbia University Institutional Review Board (IRB-AAAL4860).

### Fish Collection

Data were collected over four days in June and July of 2013. Nine sites were selected within and outside the fringing reef adjacent to Nagigi village (Figure 1, Table 1). Fish specimens were collected using spearfishing and the application of the fish anesthetic MS-222, following Columbia University IRB protocols (Proposal IRB-AAAF6300). Additional species were observed on the reef but not sampled due to their conservation status or a lack of sampling opportunity. After collection fish were photographed and identified to species level using field guides [21]. Gill or muscle tissue was collected and stored in liquid nitrogen, while whole specimens were preserved in formalin and have since been accessioned to the collection of the American Museum of Natural History, New York.

**Table 1:** Sampling locations with latitude and longitude.

Location #	Site Name	Latitude	Longitude
1	FJ_01	s 16d 48.39″	e 179 28.474″
2	FJ_02	s 16′ 48.608″	e 179′ 28.545″
3	FJ_03	s 16° 48.451′	e 179° 28.138′
4	FJ_04	s 16′48.377″	e 179′ 28.757″
5	FJ_05	s 16′ 48.323″	e 179. 28.554″
6	FJ_06	s 16′ 48.409″	e 179′ 28.594″
7	FJ_07	s 16′ 48.340″	e 179′ 28.598″
8	FJ_08	s 16′ 48.667″	e 179′ 28.602″
9	FJ_09	16° 48.180′S	179° 28.738′E

### Fisher Interviews

Twenty-two individual fishers were interviewed singly and in groups across a total of 15 interviews. Interviews followed an IRB-approved questionnaire and participants' written consent was obtained in accordance with IRB protocol (Proposal IRB-AAAL4860). Most interviews took place in participants' homes, but some occurred while participants fished or gleaned on the reef at low tide and one interview took place during a kava-drinking session in front of the village hall. Interviews were generally about half an hour long, but some were considerably shorter or longer depending on context, language barriers, and participants' knowledge. For instance, one interview in which participants knew little to no English lasted only ten minutes, while the kava-drinking session lasted more than two hours. Three native Fijian speakers assisted the researchers as translators.

Of the participants, eight were male and 14 were female, and participants' average age was 50. We deliberately sought out older, expert fisherwomen because their knowledge has typically been discounted in studies of traditional ecological knowledge in the Pacific region, despite their important contribution to subsistence fisheries [22]. Participants had an average of 44 years' experience fishing on Nagigi's reef, ranging from one woman who had moved to the village a year ago to three villagers in their sixties who had lived in Nagigi their whole lives and presumably fished and gleaned on the reef from a young age. We deliberately chose participants who varied greatly in age and experience in order to gain impressions of fishers' knowledge across generations and experience levels.

## Results

### Partial Species List

In total, 150 species were recorded on Nagigi's reef (Table S1). Of these, 96 (64%) were collected and accessioned to the American Museum of Natural History in New York and 15 (10%) were sighted by researchers but not collected. Sixty-three were mentioned in interviews by expert fishers. Seventeen species were both collected and mentioned in interviews (11.3% of total species collected). Fijian names were recorded for 82 species. Several additional generic Fijian names were recorded based on interviews (Table 2). The most species-rich families were Pomacentridae (16.7%) and Labridae (10.7%).

**Table 2:** Generic Fijian fish names.

Fijian name	English definition	Families represented
*baludawa*	parrotfish	Scaridae
*boila*	moray eel	Muraenidae
*dridri*	several surgeonfishes	Acanthuridae
*kake*	several snappers	Lutjanidae
*kawakawa*	grouper	Serranidae
*labe*	several wrasses	Labridae
*lematua*	scorpionfish	Scorpaenidae
*nuru*	any small finger-length fish	Apogonidae, Pomacentridae, etc.
*rawarawa*	blue or green parrotfish	Scaridae
*rusarusa*	small rabbitfish	Siganidae
*sumusumu*	pufferfish	Tetraodontidae
*tivitivi*	butterflyfish	Chaetodontidae
*ulavi*	gray or white parrotfish longer than 30 cm	Scaridae

Several generic Fijian names (*i.e.* those which represent multiple species) with approximate scientific definitions.

### Social Characteristics of the Nagigi Fishery

**Figure 2:**
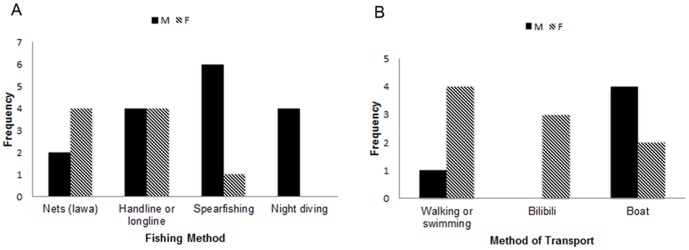
Fishing and transport methods. Frequency with which common fishing and transport methods were mentioned by men and women in 15 interviews.

Across all 15 interviews, male villagers were more likely to describe spearfishing and night diving as their main activities, while female villagers were more likely to use fishing nets. Men and women both used fishing lines frequently. Men fished from boats more frequently, while women were more likely simply to walk or swim to the portion of the reef on which they planned to fish. Only women used *bilibili*, or traditional bamboo fishing rafts (Figure 2).

Most interviewees said they fished daily or at least once a week. The majority (11 out of 16) of participants said that they fished only for their own home consumption or for some combination of personal consumption and cash income. Three young men all mentioned selling fish, octopus, and bêche-de-mer (sea cucumber) at the market in Savusavu, the nearest town, as a source of “quick cash” (Interview 7). One couple have a small business selling fish at the Savusavu market to gain the income they need to maintain their house and educate their children. As the husband put it, “our bank is in the sea” (Interview 3). According to his wife, he is known in the village as an expert fisherman who can reliably bring home fish the length of a forearm (about 40–50 cm), unlike the rest of the village's fishermen, who only catch hand-length fish (about 20 cm). One 52-year-old woman, who is a community-acknowledged expert octopus fisherwoman, sells octopus and forearm-length fish at the market in Savusavu and keeps smaller fish for her own consumption (Interview 9). Another woman says that she fishes for her and her husband's consumption and to sell at market but also to share with her friends and fellow villagers: “anybody here wants to eat fish, they just come.I just keep a little bit for the two of us” (Interview 8).

Interviews indicate that fishing labor is primarily divided along gender lines, and that it is a frequent occupation for both men and women. There is no clear divide between those who fish for income (artisanal fishers) and those who fish for their own consumption (subsistence fishers); rather, villagers pursue both options as they need protein or cash. Fishing also appears to facilitate social relationships, as with the sharing of fish among friends and relatives, and upward socioeconomic mobility, as demonstrated by the couple who use their fishing income to pay their children's school fees.

### Identifying At-Risk Species

**Figure 3:**
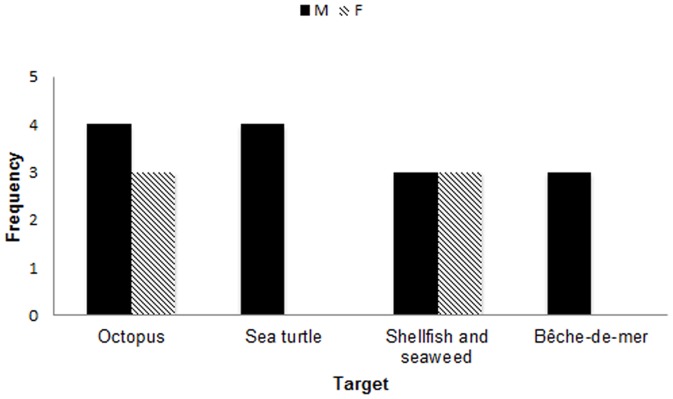
Non-finfish targets of men and women's fishing. Sea turtle and bêche-de-mer harvesting are primarily male pursuits while reef gleaning (for octopus, shellfish, seaweed, etc.) is more egalitarian.

The most common species that participants reported targeting was *kuita* (octopus *Octopus spp.*), with *kabatia* (thumbprint emperor, *Lethrinus harak*), *kanace* (bluetail mullet, *Moolgarda engeli*) and *saqa* (giant trevally, *Caranx ignobilis*) only slightly less in demand (Table 3). Octopus fishing for market seems to be a primarily, though not exclusively, female skill, passed on from grandmothers to their children and grandchildren. Several non-finfish targets were mentioned repeatedly during my interviews, including octopus (*kuita*), sea turtle (*vonu*), shellfish, and bêche-de-mer (Figure 3). Only men described targeting turtle and bêche-de-mer, while the general category of reef gleaning, which includes the targeting of octopus, shellfish, and seaweed, was a more egalitarian pursuit. The bêche-de-mer fishery serves exclusively as a source for cash income in Fijian society, and no participants reported eating sea cucumbers. Holothurians are typically caught and dried in salt and then traded with foreign vessels for entry into Asian food markets. In contrast, turtle (*vonu*) meat is consumed in Fijian villages on celebratory occasions, and thus the fishery is largely driven by local demand and culture.

**Table 3:** At-risk reef species.

Fijian Name	Scientific Name	Number of Times Mentioned	Perceptions of Pop. Change (n = number of participants with this perception)
Kuita	*Octopus* sp.	8	Decreasing size (n = 1) and abundance (n = 3)
Kabatia	*Lethrinus harak*	6	Decreasing abundance (n = 1)
Saqa	*Caranx ignobilis*	6	Decreasing abundance (n = 1)
Kanace	*Moolgarda engeli*	6	Smaller, scarcer, and harder to catch (n = 1)
Ulavi	Gray or white parrotfish larger than 30 cm	5	Increasing abundance (n = 1)
Vonu	Sea turtles	5	Decreasing abundance (n = 2)
Labe	*Halichoeres trimaculatus*	5	N/A
Nuqa	*Siganus vermiculatus*	5	Decreasing abundance (n = 1) or increasing abundance (n = 1)
Kawakawa	*Epinephelus polyphekadion*	4	Decreasing size and abundance; increased fishing effort necessary (n = 5)
Ta	*Naso unicornis*	4	N/A
Tabace	*Acanthurus triostegus*	4	N/A
Dridri	3 *Acanthurus* sp.	4	Increasing abundance (n = 1)
Vasua	*Tridacna gigas* (sea clams)	4	Decreasing abundance (n = 1)
Deou	*Upeneus vittatus*	4	N/A

Most at-risk reef species based on the number of villagers who claimed to target them. Includes perceived changes in the population of these species and the number of interviewees who made these assessments.

### Villagers' Perceptions of Reef Change

Nearly all participants said that fish size and abundance had decreased over the years they'd lived in Nagigi and that the required fishing effort had increased (Table 4). Participants described having to go farther or work longer to catch enough fish. In particular, villagers said that *kawakawa* (a generic Fijian name for small species of groupers, genera *Epinephelus* and *Cephalopholi*; Serranidae) and octopus had decreased in size and abundance. As one villager put it, “Before, they used to catch eight, nine [octopus] sometimes. But now—you can just catch two, three” (Interview 7). Several participants said that the boxfish (*Ostracion cubicus*) had disappeared from the reef entirely. *Varivoce* (humphead wrasse or *Cheilinus undulatus*) and *kalia* (bumphead parrotfish, *Bolbometopon muricatum*) have also become scarce. These findings echo range-wide population dynamics in these species: *C. undulatus* is listed as endangered on the IUCN Red List and *B. muricatum* is categorized as vulnerable [23], [24] Two villagers reported that *nuru* (any fish shorter than a finger's length) have become scarce and that inshore coral heads, which are an important source of habitat for the *nuru*, are dead or degraded.

**Table 4:** Villager perceptions of reef change across 12 interviews.

Interview #	Average Years in Village	Perceptions of Reef Dynamics
1	58	Populations of *kabatia, kawakawa* and *ululoa* have decreased. Populations of *balagi, nuqa, dridri*, and *ulavi* have increased; interviewee perceived these as coming from outside the reef. Interviewee has seen no change in her octopus catch because she is an expert octopus fisherwoman and knows where the breeding ground is.
2	49	*Nuru* have disappeared and inshore corals are mostly dead. Fish abundance has decreased. In particular, *kawakawa* and *ose* have decreased in abundance and become smaller.
3	50	*Varivoce* and *kawakawa* have become harder to find.
4	52	Boxfish and sea prawns have become hard to find.
5	62	*Nuqa* is hard to find. Sea crabs and sea prawns aren't abundant.
6	66	Fish abundance has decreased.
7	34	Fish size has decreased. *Toto* have disappeared. There used to be lots of *kalia*, but now they are scarce. Octopus are smaller and scarcer: “Before, they used to catch eight, nine sometimes, but now—you can just catch two, three.” *Balagi* and *ogo* are plentiful. It's still possible to catch large *kawakawa*, but fishers have to go outside the reef.
8	More than 30	Fish abundance and size have decreased. Villagers have to go farther outside the reef to go fishing and put in more effort to catch the same amount of fish.
9	52	Octopus have become scarce.
10	About 2	Fish have decreased in abundance. *Kanace, vonu*, and *kawakawaloa* used to be more abundant and easier to catch close to shore when the interviewee was growing up in Nagigi (about 30 years ago).
11	60	Fish don't come in close to the shore anymore, so they're harder to catch. *Nuru* are hard to find and the coral is mostly dead. Fish are smaller. *Ogo* and snapper in particular don't come in close to the shore. *Saqa, saku, yawa, vilu, vonu, vasua*, and octopus are scarce.
12		*Varivoce* are scarce. In 1998, Fiji had a very hot season that noticeably raised the temperature of the ocean, causing the coral to die. Since then the coral has been recovering from that event. There is a quarry on Nagigi Creek upstream from the village that flushes sediment and waste down to the shore, killing the coral. A government research station along the creek uses weedkiller, which travels down to the sea when it rains and kills the coral. In 2001, the village held a traditional 3-month *tabu* (fishing moratorium) to honor the memory of a chief who had died.

See Table S1 for translations of Fijian species names.

Three villagers cited the smashing of inshore coral heads while fishing, either purposefully or accidentally, as one cause of this habitat loss and fish population decrease. Interestingly, both members of one married couple mentioned this issue in separate interviews, but the husband described coral-smashing as a deliberate fishing technique while his wife believed that it occurs accidentally when fishers walk on the reef. (Interviews 8, 11).

Nagigi's *Turaga ni Koro* (elected village headman) proved to be an especially valuable source of information about environmental change. Although he no longer fishes, his role as headman requires him to act as a liaison between Nagigi Village and the Savusavu provincial office and to represent the village's interests. He also prosecutes poachers from outside Nagigi, people who fish without a license, and those who violate laws such as the bans on turtle fishing, use of the poisonous *duva* root, and targeting endangered species. Perhaps as a result, he proved highly knowledgeable about and observant of reef dynamics. He cited three sources of environmental stress that no other villagers mentioned: (1) development inland of Nagigi that flushes sediment and pesticides down Nagigi Creek to the reef, (2) an unusually hot season in 1998 that placed temperature stress on the coral, and (3) demand for prawns from nearby resorts that has spurred overharvesting of these organisms.

### Conservation Attitudes and Suggested Solutions

Interviewees unanimously supported the establishment of a marine protected area or *tabu* on Nagigi's reef. None of them, however, believed that an MPA should last permanently or should extend over the village's entire fishing grounds. Suggestions ranged from protections lasting one year to ten years. The *Turaga ni Koro*, who has been a vocal proponent of the MPA plan, believes that a closure should last for five years. Another participant thinks that only a one-year MPA is necessary, while his wife thinks it should be “the longer the better” (Interview 8). Three young men who fish to make “quick cash” believe that a three-year closure would be enough for most fish, including the IUCN red-listed *Bulbometopon muricatum*, to regain their former size. One interviewee had recently moved to Nagigi from a neighboring village, which had just decided to extend its five-year *tabu* for another five years after observing rebounding fish populations, and she was consequently very supportive of Nagigi's plan.

No interviewees expressed concern about losing their fishing income or subsistence catch while the MPA was in place. Several spoke about their apprehension for the future if conservation steps were not taken. One man expressed the expectation that the *tabu* area would provide a safe breeding area and would “overflow with fish,” increasing the villagers' catch throughout their marine tenure area (Interview 11). One couple that moved to Nagigi two years ago had not heard of the plan to create an MPA, but when the project's purpose was explained to them, they agreed that it would be a good idea because they had noticed that Nagigi's fish populations were scarce. Only one interviewee expressed reservations about the MPA plan because she noticed that when a neighboring village set a *tabu* area on its reef, the villagers came to Nagigi's reef at night to fish clandestinely. She thinks that if an MPA is set up in Nagigi, the village's fishermen will do the same on neighboring fishing grounds.

## Discussion

### Fishing as a Way of Life

Our results highlight the way that fishing and reef gleaning possess a social significance in the political ecology of villagers of Nagigi beyond simply providing a source of protein and cash. Few of our interviewees reported that they did not fish at all, and those who fished infrequently usually qualified this by saying that they were “too old” or were pregnant or nursing, the latter of which groups is subject to strict food prohibitions [25]. This suggests that fishing is a basic way of life in Nagigi, but may wax and wane over villagers' life spans as they pass through various life stages such as motherhood and old age. Fishing also provides social and recreational benefits as well as economic ones, with some participants describing fishing as their hobby or saying that they loved to catch certain fish (Interview 8). Jones [6] notes a similar enjoyment of fishing among the Fijian fisherwomen in the Lau archipelago who were her ethnoarchaeological study subjects, and notes that Fijian fishing expeditions reflect cultural customs and social relations as well as the need for food and income.

Two married couples described fishing together for personal consumption or as part of a small business, and one man said that his wife had taught him how to fish with a handline, an unusual statement in a society in which teaching is usually vertical (i.e., parents to children) (Interview 11) [26]. There seems to be a significant inter- and intra-village trade in fish that is informal and based around relationships with family and friends. As one villager put it, “if I don't eat it, I'm happy to just give it out to others” (Interview 8). Another, a recent transplant to Nagigi from a neighboring village, told me that she does not fish at all, but that when she wants fish, she asks her brothers at home to bring her some (Interview 15). Because no cash exchanges hands, or does so only within the village, such trade necessarily is included in assessments of Fiji's artisanal fishery, complicating fisheries assessments and making management more difficult.

### Conserving At-Risk Species

**Figure 4:**
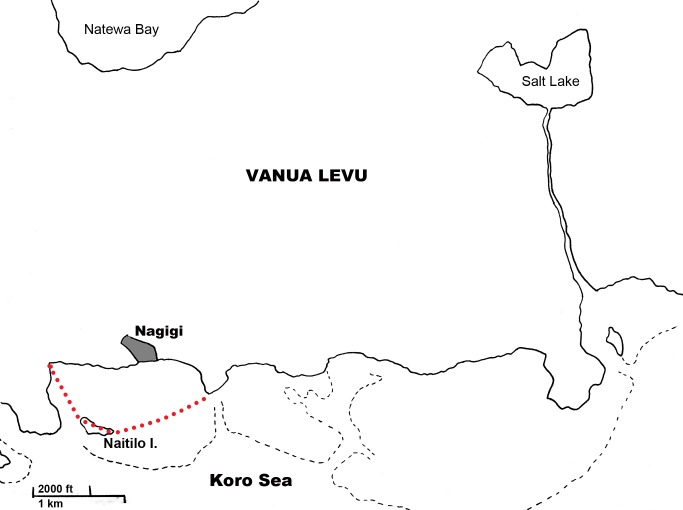
Map of Nagigi showing proposed MPA site. The proposed MPA includes 1^2^ of reef flat and seagrass meadow directly in front of Nagigi village. It excludes the mangrove swamp to the east of the village and any area outside the fringing reef. Black dashed lines show fringing reefs, while red dotted line shows marine protected area site proposed by the *Turaga ni Koro*.

The proposed marine protected area in Nagigi would consist of one square kilometer of intertidal habitat directly in front of the village (Figure 4). The proposed area includes some fringing reef, patch reef and seagrass beds. The inclusion of this diverse set of habitats promises to strengthen the proposed reserve, as it accounts for ontogenetic changes in habitat use by many species, including *L. harak* [27]. However, Jupiter and Egli [15] suggest that MPAs should be at least 1400 m on a side to make sure that they are larger than the home ranges of targeted species. *Lethrinus* spp., for instance, can move up to 700 m, and do so mostly at night, making them vulnerable to poaching and night fishing. Aswani and Hamilton [28] recommend that small reserves should be 4–6 km in diameter to be effective. And while small reserves (<1 km^2^) can be effective, more recent research suggests that large size is a strong indicator of reserve success [29], [30]. Based on these recommendations, it seems unlikely that Nagigi's MPA will have a significant positive effect on reef fish populations without a twofold or threefold increase in size.

Even short-term MPAs are known to increase abundance and biomass of targeted species, leading to increased recruitment and migration of fish into neighboring reefs, but it is unclear how long an MPA of the size of Nagigi's would need to last in order to make these benefits tangible [15], [17]. Several of the species most heavily targeted by Nagigi's villagers do not reach sexual maturity until the age of three or four years, suggesting that a three-year MPA would not be enough to ensure that an entire reproductive cohort could reach maturity without facing fishing pressure (Table 5). In *Lethrinus harak*, for instance, which is one of the most heavily targeted fish in Nagigi, females reach maturity within one or two years [31]. However, *L. harak* is a protogynous hermaphroditic species, and females begin to transition to males at age three or four [31]. In addition, *L. harak* utilizes both coral reef and seagrass habitats at different life stages, indicating that both habitat types are critical for *L. harak* conservation [27]. Based only on female maturation rate, and without taking this habitat use into account, one would anticipate that a three-year MPA would be enough to increase local populations of the species, but greater knowledge of *L. harak*'s life history and reproductive strategy reveals that a longer period and larger area of protection are needed.

**Table 5:** Age at maturity of five of the most frequently fished species in Nagigi, previously identified in Table 3.

Fijian Name	Scientific Name	Age at first spawning
Kabatia	*Lethrinus harak*	Protogynous hermaphroditic; females reach sexual maturity at 1–2 years and transition to males beginning at age 3 or 4 [31]
Saqa	*Caranx ignobilis*	Reach maturity at about 3.5 years [46]
Vonu	*Chelonia mydas*	Estimated 30 years [47]
Nuqa	*Siganus vermiculatus*	Females: 1 year or younger [48]
Deou	*Upeneus vittatus*	Total life span 3 years; estimated age of sexual maturity 1 year [32]

Includes four finfish and one sea turtle species, ranked by intensity of fishing pressure (highest to lowest).

Similarly the local population of *Upeneus vittatus*, another heavily targeted species, would benefit from a three-year MPA. *Upeneus vittatus* reaches maturity within a year and has a total life span of three years [32]. However, Nagigi's *U. vittatus* population is catadromous and highly geographically specific in its habitat use, traveling once a year from its habitat in Navatu Lake, northeast of Nagigi, to the mangrove swamp west of the village to spawn (Figure 4). This mangrove swamp is outside the proposed marine protected area and the fish face significant harvesting pressure from villagers during their spawning period, which occurs October through December. This harvesting event is invested with cultural significance in Nagigi, and the entire population of the village dresses up in fine clothes and garlands, known as *salusalu*, to harvest the spawning fish. Villagers believe that they must not use knives to gut *U. vittatus* during spawning season, or the fish will never return to their spawning site, and that they must not sell the fish but instead share them with those who are less fortunate (Interviews 7, 8). Although no villagers expressed concern about declines in the abundance or biomass of *U. vittatus*, these traditional beliefs and respect for the spawning aggregation could motivate village leaders to place restrictions on fishing this species if population levels begin to show signs of decline.

These recommendations about MPA size are predicated on the assumption that fishing effort would be dispersed on either side of the reserve. However, Cinner [33]–[35] points out that in some traditional fisheries, when alternative employment opportunities exist, some people opt out of the fishery altogether when reserves are established. For example, one could imagine a scenario in which fishers from Nagigi switch from fishing to farming should the costs of fishing in the non-reserve area become too high. Although these switching behaviors can be couched in terms of optimal foraging theory—namely, that the energetic and time costs of traveling farther to reach the fishery would outweigh the energetic or monetary benefits of fishing—we urge caution in interpreting people's activities strictly through the lens of optimal foraging. Although useful, this theory runs the risk of ignoring cultural and social aspects of fishing that are not captured in such models.

### Perceptions and Realities of Reef Change

In general, villagers recognized an interlocking complex of economic causes as being primarily responsible for the overfishing of Nagigi's reefs. According to interviewees, the village's population has grown in recent decades as the cost of living in Fiji has increased, leading to increasing numbers of villagers fishing intensively on the reef for cash income (Table 6). The bêche-de-mer fishery and the practice of night fishing, in particular, draw village youth in search of “quick cash” because bêche-de-mer commands high prices on the foreign market and night diving yields higher catch per unit effort than lower-tech day diving (Interview 7). One interviewee reported that in a single hour of night fishing he could expect to catch 10 kg of fish because the fish are “not using as much energy, because they are sleeping the spear, you can put it closer to the head” (Interview 7). Johannes [18] observed a similar strategy among fishermen in Palau.

**Table 6:** Perceived causes of reef dynamics over participants' lifetimes, by frequency with which they are cited and participants who cited them.

Cause of Environmental Change	Number of Mentions	Mentioned by:
Increase in fishing pressure for market instead of subsistence, especially for “quick cash”	9	P#1, 2, 3, 4, 10, 14, 16, 19, 21
Poaching by outsiders	6	P#4, 5, 6, 7, 19, 20
Increasing population of Nagigi	3	P#1, 15, 17
Coral smashing either as a fishing method to flush out *nuru* or by accident while walking on the reef	3	P#3, 15, 19
Night fishing	3	P#13, 19, 20
Profitability of bêche-de-mer fishery and toxicity of injured holothurians	3	P#4, 19, 20
Increased cost of living	2	P#19, 20
Changing climate patterns and sea level rise	1	P#1
Demand for sea prawns from local resorts	1	P#20
Unusually hot season in 1998 which placed stress on coral ecosystem	1	P#20
Ongoing upstream development flushing sediment and weedkiller onto reef	1	P#20
Use of *duva* root as fish poison	2	P#1, 19
Use of nets with small openings that catch juveniles	1	P#21

Note that a few highly knowledgeable participants (#19, #20) provided the source for many observations.

**Figure 5:**
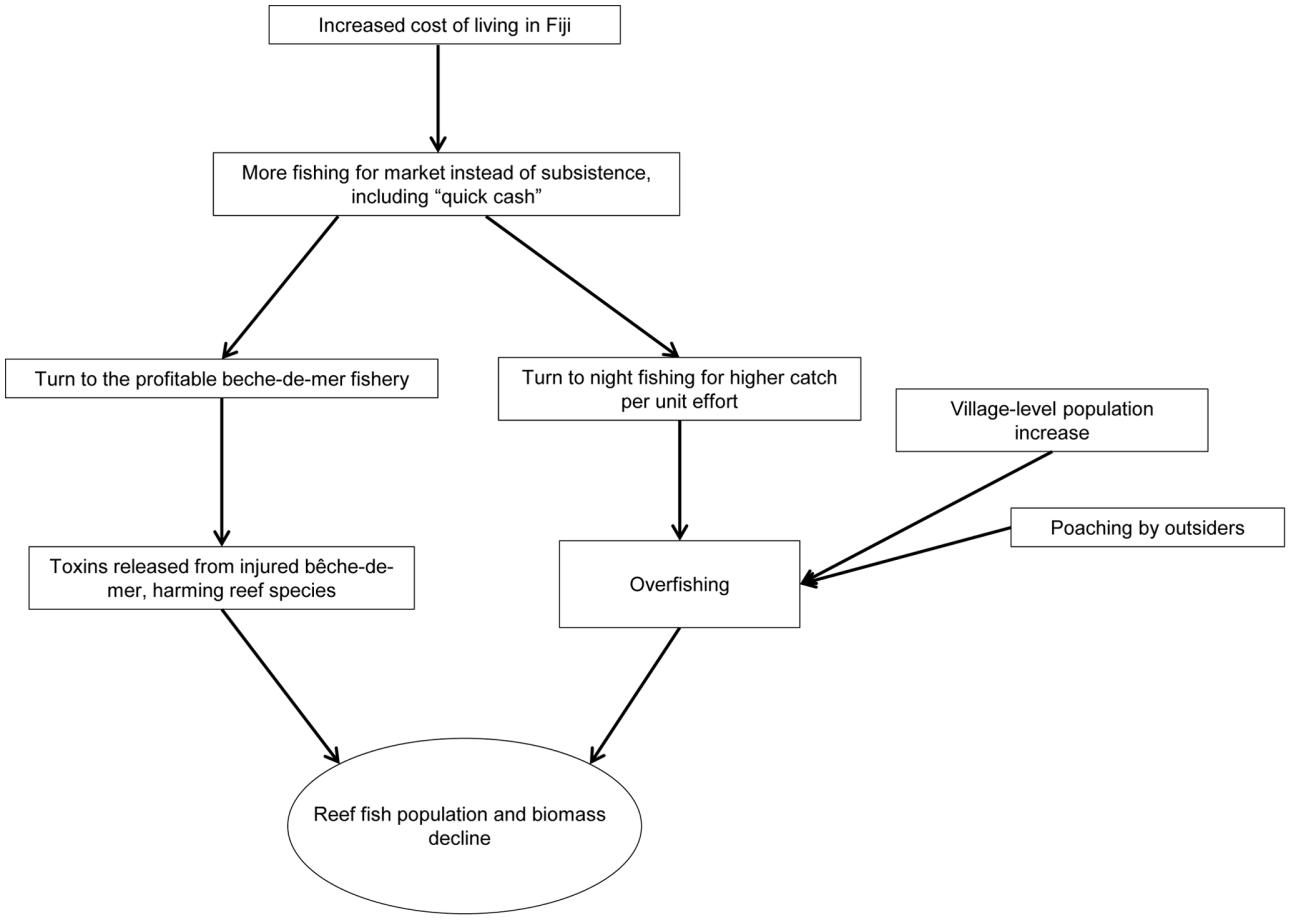
Economic and social causes of fish population decline. Flowchart representing the causal relationships between the economic and social factors to which villagers attribute fish population and biomass decline.

Two participants expressed particular worry about the increase in the bêche-de-mer harvest because holothurians release toxins when injured or killed that can harm reef fish; in fact, they have a well-documented history of use by indigenous societies in the South Pacific as a fish poison similar to *duva* (*Derris* spp., Fabaceae) root [36]. One participant said that he personally did not harvest bêche-de-mer because he “knows their role in the ocean,” alluding to their importance in bioturbation [37]. Out of 15 interviews total, in nine of them the participants attributed overfishing to some part of this combination of the cost of living, the village's population increase, the desire for a “quick catch,” the profitability of bêche-de-mer, and night fishing (Figure 5). This increasing reliance on the bêche-de-mer fishery for cash income is especially worrisome from an economic standpoint because holothurian populations are likely to decline as reefs degrade due to the impacts of ocean acidification and climate change [38].

It is difficult to correlate villagers' perceptions of reef change with quantitative measures of reef fish population dynamics because such data is scarce and patchy for the Pacific region [39]. However, regional- and national-level fisheries reports bear out villagers' perception that finfish abundance and biomass are in decline in Fiji, although data is not available for the island of Vanua Levu in particular. Richards *et al.* [39] note a 16.5% decrease in Fiji's artisanal finfish production by weight between 1988 and 1992, implying that finfish biomass and abundance decreased over this period. A general trend of population growth is well-documented in the South Pacific and demand for fisheries products is expected to remain high, exacerbating the problems of overfishing that villagers have perceived [40].

### Understanding Conservation Attitudes in Nagigi

A number of participants, when asked if Nagigi had ever had a *tabu* before during their lifetimes, mentioned the traditional reef closure for the death of a chief. According to this tradition, after a village chief dies, the fishing ground is closed for a hundred nights so that the fish population will increase. The start of the *tabu*, according to one account, is signified when members of the chief's funeral party come down to the reef after his burial and wash their hands in the sea. After the hundred nights, fishing begins again, and by this point the temporary closure has allowed local fish populations to increase enough that the villagers can fill two boats with fish in honor of the chief. These fish are divided into two parts and shared between the chief's *mataqali*, or clan, and the rest of the village (Interview 1). Such a save-and-spend model for marine closures is well established in Fijian culture and is known to have predated Western contact [41], [42]. Nagigi last held a *tabu* for the death of a chief in 2001 (Table 4).

Any MPA project in Nagigi designed explicitly for biodiversity conservation will have to take into account the prevailing Fijian notion that closure areas are a kind of short-term “food bank” that can be opened and closed at will. Leaving aside the question of whether a three-to-five-year MPA will provide enough time for populations to rebound, as discussed above, it seems reasonably likely that Nagigi's villagers will decide to open the protected area for a short period at some point during the closure to pay for special projects or to feed visitors. Several interviewees stated explicitly that they think a future MPA should be opened if a chief dies or to if they have to prepare a feast for special visitors (Interviews 1, 9). Previous research in Fiji has shown that these project-specific openings, however brief, can have significant negative impacts on fish populations and can become a seductively convenient resource once villagers see how profitable they can be [15], [41]. In a case documented by Jupiter *et al.* [42], inhabitants of Fiji's Kia Island collectively decided to suspend their local marine closure for a few days in order to raise money for community projects. Originally, the islanders aimed to raise FJD$12,000 (about US$7,500), but when they exceeded this goal on the first day of the fundraiser, they elected to continue the harvest. Residents fished in shifts for 24-hour periods six days a week during the five weeks, and netted an estimated FJD$200,000. Jupiter *et al*. [42] observed significant loss of fish biomass in large-bodied species such as acanthurids, carangids, and scarids during the harvest event, with negative effects lingering a year later. When presenting management options to village leaders in Nagigi, it is important to note these findings and warn them that marine protected areas may not have the desired effect of increasing fish populations if the MPA is opened to intense fishing pressure for even short periods of time.

One possible strategy to counter this spend-and-save effect is to extend some species-specific prohibitions beyond the term of MPA protection. For instance, long-term fishing protection for the endangered *Bolbometopon muricatum* and other similarly vulnerable species may offer lasting conservation benefits [25], [43]. Precedent for such species-specific bans exists in Fijian culture in the form of traditional *tabus* that forbid certain groups from consuming specific species. For example, Nagigi contains a large demographic of Seventh Day Adventists, who do not consume or harvest any marine species that do not possess fins and scales (Interviews 8, 11). This means that they do not contribute to the harvesting pressure for sea turtles, clams, octopus, and other shellfish. The villagers of Nagigi also possess the cultural belief that the consumption of lizardfishes (Synodontidae), known as *dolo*, will render men impotent (Interview 7). As a result, the men of Nagigi will not eat lizardfish, though women will. And, throughout Fiji, *tabus* exist against women's consumption of various marine species during pregnancy and lactation, which help protect women and their infants from life-threatening ciguatera poisoning [25]. All of these existing models could lead to cultural acceptance for a species-specific ban designed to relieve overharvesting pressure.

While several MPAs have been created in Fiji with the tourism sector in mind, Nagigi's villagers have been silent on using ecotourism as a way to monetize their proposed MPA [44], [45]. Several large dive resorts exist within 15 km of Nagigi and there is an airport approximately 20 km away in Savusavu, so the region has a well-developed tourism capability. However, creating Nagigi's MPA independent of any attempt to attract ecotourism could serve to insulate the reserve and Nagigi's villagers from dependence on a volatile global economy.

### Sources of Bias

The list of fish sampled for this paper should not be taken as a complete representation of the location's biodiversity due to the extremely short period of sampling and flaws in the sampling methods used. Fishes with an interstitial coral habitat, including the speciose groups Gobiidae and Blenniidae, are particularly underrepresented due to strong currents on sampling days meant collection of these groups with MS-222 was impractical. Although further sampling efforts will be necessary to determine the overall biodiversity of Nagigi's reef habitat, species targeted by local fishers are well-represented in the collection, making it a valuable if incomplete survey.

The interview-based portion of this study is also subject to limitations due to the brief survey period and small sample size. With only 22 participants, it is impossible to tell which beliefs about reef dynamics are common or unusual within the village, and the contributions of a few social demographics, especially the youth of the village, are lacking. The language barrier meant that some of questions may have been unclear to participants, and some of the nuances of their responses were undoubtedly lost in translation. The *Turaga ni Koro* facilitated introductions to 16 out of 22 of interview subjects. In most cases he conducted the researcher to participants' homes and either translated or waited outside while the researcher worked with another translator. In several instances, he interjected his own knowledge and opinions into the conversation when he sensed that a point needed clarification. He is well informed about village and regional issues, has lived in Nagigi for most of his life, and has been a vocal proponent of the plan to create a marine protected area on Nagigi's reef. Due to this advocacy, his choice of participants might have meant that we spoke mostly with villagers who were also supportive of the MPA idea. However, interviewees who were approached without the *Turaga ni Koro's* mediation also expressed strong support for the MPA project. Interviewees may also have been unwilling to mention that they targeted turtles, sharks, and other protected species such as *B. muricatum* for fear of prosecution for violating Fijian law.

## Conclusions and Implications

Based on its limited temporal and geographic scope, this study necessarily deals with only a small group of marine management stakeholders and makes conservation recommendations for only a small area of Fiji's coastline. However, we believe the synthesis of biodiversity sampling and fisherman interviews described here provides a template for future research projects that seek to integrate the traditional ecological knowledge of indigenous communities into conservation planning. Members of indigenous fishing communities can provide information about changes in reef health over time, the socioeconomic uses of marine resources, and the population dynamics of at-risk reef species—data that can be difficult and time-consuming to gather using conventional means, especially in developing countries. The necessity of gathering such information is highlighted by the fact that, in this study, only 11.3% of total species were captured by both methods of data collection. Without using both destructive sampling and interview techniques, a significant amount of data about the composition of Nagigi's reef ecosystem would have been lost for the purposes of this study. But, by combining these two methods, researchers can construct a clearer and more complete picture of the reef ecosystem and fishers' needs. Based on this information, researchers can then tailor their conservation recommendations to the life histories and habitat needs of targeted species, while taking into account the subsistence needs of local communities.

## Supporting Information

Table S1 Reef Fishes of Nagigi. Partial list of the reef fishes of Nagigi by order and family, with common names and local Fijian names (if available). Species accessioned to the AMNH collection are in bold. C = collected, I = interview, S = sighted but not collected.
(DOCX)
