# Correction: Combining Natural History Collections with Fisher Knowledge for Community-Based Conservation in Fiji

**DOI:** 10.1371/journal.pone.0101407

**Published:** 2014-06-20

**Authors:** The PLOS ONE Staff

## Notice of Republication

This article was republished on June 5, 2014, to remove copyrighted information. Please download this article again to view the correct version. The originally published, uncorrected article and the republished, corrected article are provided here for reference.

## Supporting Information

File S1 Originally published, uncorrected article.
(PDF)

File S2 Republished corrected article.
(PDF)
