# Supplementary material for: Combining Natural History Collections with Fisher Knowledge for Community-Based Conservation in Fiji
Source: PLoS One. 2014 May 21;9(5):e98036. doi: 10.1371/journal.pone.0098036 (PMC4030001; doi:10.1371/journal.pone.0098036)
Supplement: Table S1 — Reef Fishes of Nagigi. Partial list of the reef fishes of Nagigi by order and family, with common names and local Fijian names (if available). Species accessioned to the AMNH collection are in bold. C = collected, I = interview, S = sighted but not collected. (DOCX) [file pone.0098036.s001.docx]

Supplementary Table 1. Reef Fishes of Nagigi

| **Scientific Name** | **Common Name** | **Fijian Name** | **Sampling** |
| --- | --- | --- | --- |
| **MYLOBATIFORMES** | | | |
| **Myliobatidae** | | | |
| *Aetobatus narinari* | spotted eagle ray |  | S |
| **RAJIFORMES** | | | |
| **Rhinobatidae** | | | |
| *Rhynchobatus sp.* | guitarfish |  | S |
| **ANGUILLIFORMES** | | | |
| **Muraenidae** | | | |
| *Echidna nebulosa* | snowflake moray | boila | S, I |
| **Ophichthidae** | | | |
| *Myrichthys colubrinus* | harlequin snake eel | dakulavi | I |
| **GONORYNCHIFORMES** | | | |
| **Chanidae** | | | |
| *Chanos chanos* | milkfish | yawa | I |
| **BERYCIFORMES** | | | |
| **Holocentridae** | | | |
| *Myripristis berndti* | blotcheye soldierfish | corocoro | I |
| *Sargocentron spiniferum* | sabre squirrelfish | dravisau | I |
| **MUGILIFORMES** | | | |
| **Mugilidae** | | | |
| ***Neomyxus leuciscus*** | **acute-jawed mullet** |  | **C** |
| *Valamugil engeli* | bluetail mullet | kanace | I |
| **BELONIFORMES** | | | |
| **Belonidae** | | | |
| *Tylosaurus crocodilus* | crocodile needlefish | saku | I |
| **TETRAODONTIFORMES** | | | |
| **Balistidae** | | | |
| ***Balistapus undulatus*** | **orange-lined triggerfish** |  | **C** |
| ***Rhinecanthus aculeatus*** | **Picasso triggerfish** | **cumu** | **C, I** |
| ***Rhinecanthus aculeatus*** | **white-barred triggerfish** | **cumucumu** | **C, I** |
| ***Sufflamen chrysopterus*** | **halfmoon triggerfish** |  | **C** |
| **Diodontidae** | | | |
| ***Diodon hystrix*** | **spotfin porcupinefish** | **sokisoki** | **C, I** |
| **Monacanthidae** | | | |
| ***Amanses scopas*** | **broom filefish** |  | **C** |
| **Ostraciidae** | | | |
| *Ostracion cubicus* | yellow boxfish | toto | I |
| **Tetraodontidae** | | | |
| ***Arothron nigropunctatus*** | **blackspotted puffer** | **sumusumu** | **C** |
| ***Canthigaster solandri*** | **spotted sharpnose** |  | **C** |
| **AULOPIFORMES** | | | |
| **Synodontidae** | | | |
| ***Synodus binotatus*** | **two-spot lizardfish** |  | **C** |
| ***Synodus variegatus*** | **variegated lizardfish** |  | **C** |
| **PLEURONECTIFORMES** | | | |
| **Soleidae** | | | |
| *Soleichthys heterorhinos* | black-tip sole | davilai | I |
| **SCORPAENIFORMES** | | | |
| **Scorpaenidae** | | | |
| ***Scorpaenodes guamensis*** | **Guam scorpionfish** |  | **C** |
| ***Scorpaenodes parvipinnis*** | **lowfin scorpionfish** |  | **C** |
| **PERCIFORMES** | | | |
| **Acanthuridae** |  |  |  |
| *Acanthurus leucoparius* | whitebar surgeonfish | dridri | I |
| ***Acanthurus lineatus*** | **lined surgeonfish** | **dridri** | **C, I** |
| ***Acanthurus nigrofuscus*** | **brown surgeonfish** | **dridri** | **C, I** |
| *Acanthurus triostegus* | convict surgeonfish | tabace | I |
| *Acanthurus xanthopterus* | yellowfin surgeonfish | balagi | I |
| ***Ctenochaetus binotatus*** | **twospot surgeonfish** |  | **C** |
| ***Ctenochaetus striatus*** | **striated surgeonfish** | **ikaloa** | **C, I** |
| ***Naso lituratus*** | **orangespine unicornfish** | **jila** | **C** |
| *Naso unicornis* | bluespine unicornfish | ta | I |
| ***Zebrasoma scopas*** | **twotone tang** |  | **C** |
| **Apogonidae** | | | |
| ***Apogon angustatus*** | **broadstriped cardinalfish** | **nuru** | **C** |
| ***Apogon fraenatus*** | **bridled cardinalfish** | **nuru** | **C** |
| ***Apogon novemfasciatus*** | **sevenstriped cardinalfish** | **nuru** | **C** |
| ***Fowleria isostigma*** | **dotted cardinalfish** | **nuru** | **C** |
| ***Fowleria vaiulae*** | **mottled cardinalfish** | **nuru** | **C** |
| ***Ostorhinchus rubrimacula*** | **rubyspot cardinalfish** | **nuru** | **C** |
| **Blenniidae** | | | |
| ***Meiacanthus oualanensis*** | **N/A** |  | **C** |
| ***Plagiotremus rhinorhynchos*** | **bluestriped fangblenny** |  | **C** |
| **Carangidae** | | | |
| *Caranx ignobilis* | giant trevally | saqa | I |
| *Trachinotus blochii* | snubnosed dart | vilu | I |
| **Chaetodontidae** | | | |
| *Chaetedon auriga* | threadfin butterflyfish | tivitivi | I |
| ***Chaetodon baronessa*** | **Eastern triangular butterflyfish** | tivitivi | **C** |
| ***Chaetodon citrinellus*** | **speckled butterflyfish** | tivitivi | **C** |
| ***Chaetodon lunulatus*** | **oval butterflyfish** | tivitivi | **C** |
| *Chaetodon meyeri* | **scrawled butterflyfish** | tivitivi | S |
| ***Chaetodon pelewensis*** | **sunset butterflyfish** | tivitivi | **C** |
| ***Chaetodon plebeius*** | **blueblotch butterflyfish** | tivitivi | **C** |
| ***Chaetodon rafflesi*** | **latticed butterflyfish** | tivitivi | **C** |
| *Chaetodon unimaculatus* | **teardrop butterflyfish** | tivitivi | S |
| ***Chaetodon vagabundus*** | **vagabond butterflyfish** | tivitivi | **C** |
| ***Heniochus varius*** | **horned bannerfish** |  | **C** |
| **Cirrhitidae** | | | |
| ***Paracirrhites arcatus*** | **arc-eye hawkfish** |  | **C** |
| ***Paracirrhites forsteri*** | **blackside hawkfish** |  | **C** |
| **Gerreidae** | | | |
| *Gerres longirostris* | strongspine silver biddy | matu | I |
| **Haemulidae** | | | |
| *Plectorhincus gibbosus* | harry hotlips | bici | I |
| **Kyphosidae** | | | |
| *Kyphosus cinerascens* | blue sea chub | sirisiriwai | I |
| **Labridae** | | | |
| ***Bodianus axillaris*** | **axilspot hogfish** |  | **C** |
| *Bodianus loxozonus* | **blackfin hogfish** |  | S |
| ***Cheilinus chlorourus*** | **floral wrasse** | **drala** | **C** |
| ***Cheilinus trilobatus*** | **tripletail wrasse** | **draunikura** | **C** |
| *Cheilinus undulatus* | humphead wrasse | varivoce | I |
| ***Cirrhilabrus punctatus*** | **dotted wrasse** |  | **C** |
| ***Cirrhitichthys falco*** | **dwarf hawkfish** |  | **C** |
| *Gomphosus varius* | bird wrasse |  | S |
| ***Halichoeres hortulanus*** | **checkerboard wrasse** |  | **C** |
| ***Halichoeres trimaculatus*** | **threespot wrasse** | **labe^[[1]](#footnote-1)^** | **C, I** |
| ***Labrichthys unilineatus*** | **tubelip wrasse** |  | **C** |
| ***Labroides dimidiatus*** | **bluestreak cleaner wrasse** |  | **C** |
| ***Oxycheilinus digramma*** | **cheeklined wrasse** |  | **C** |
| ***Pseudocheilinus hexataenia*** | **sixline wrasse** |  | **C** |
| ***Stethojulis bandanensis*** | **red shoulder wrasse** |  | **C** |
| ***Thalassoma hardwicke*** | **sixbar wrasse** |  | **C** |
| **Leiognathidae** | | | |
| ***Leiognathus fasciatus*** | **striped ponyfish** |  | **C** |
| **Lethrinidae** | | | |
| *Lethrinus atkinsoni* | Pacific yellowtail emperor | sabutu/ ululoa | I |
| ***Lethrinus harak*** | **thumbprint emperor** | **kabatia** | **C, I** |
| *Lethrinus olivaceus* | longface emperor | dokonivudi | I |
| ***Monotaxis grandoculis*** | **humpnose big-eye bream** | **bu** | **C, I** |
| **Lutjanidae** | | | |
| ***Lutjanus argentimaculatus*** | **mangrove red snapper, mangrove jack** | **damu** | **C, I** |
| *Lutjanus bohar* | twospot red snapper | bati | I |
| *Lutjanus ehrenbergii* | blackspot snapper | kake | I |
| *Lutjanus fulviflamma* | dory snapper | kake | I |
| *Lutjanus fulvus* | blacktail snapper | dadreu/kake | I, S |
| ***Lutjanus gibbus*** | **humpback red snapper** | **bo** | **I** |
| *Lutjanus malabaricus* | Malabar blood snapper | rosinbogi | I |
| ***Lutjanus monostigma*** | **one-spot snapper** | **kake** | **I , C** |
| *Lutjanus russelli* | Russell's snapper | kake | C, I |
| ***Lutjanus semicinctus*** | **black-banded snapper** |  | **C** |
| *Pristipomoides sp.* | jobfish genera | pakapaka | I |
| **Monodactylidae** | | | |
| ***Monodactylus argenteus*** | **silver mono** | **koko** | **C, I** |
| **Mullidae** | | | |
| *Mulloidichthys flavolineatus* | yellowstripe goatfish | ose | I |
| ***Parupeneus barberinus*** | **dash-and-dot goatfish** |  | **C** |
| *Parupeneus indicus* | Indian goatfish | cucu | C, I |
| ***Parupeneus multifasciatus*** | **manybar goatfish** |  | **C** |
| ***Upeneus vittatus*** | **striped goatfish** | **deou** | **C, I** |
| **Nemipteridae** | | | |
| ***Scolopsis bilineatus*** | **two-lined monocle bream** | **matauka** | **C** |
| **Pinguipedidae** | | | |
| ***Parapercis clathrata*** | **latticed sandperch** |  | **C** |
| *Parapercis hexolphthalma* | speckled sandperch | dolo | C, I |
| **Polynemidae** | | | |
| *Polydactylus sexfilis* | sixfinger threadfin | ucruka | I |
| **Pomacanthidae** | | | |
| *Centropyge bicolor* | **bicolor angelfish** |  | S |
| *Centropyge bispinosa* | **twospined angelfish** |  | S |
| *Pygoplites diacanthus* | regal angelfish |  | S |
| **Pomacentridae** |  |  |  |
| *Abudefduf septemfasciatus* | banded sergeant | dumu | I |
| ***Abudefduf sexfasciatus*** | **scissor-tail sergeant** |  | **C** |
| ***Abudefduf sordidus*** | **blackspot sergeant** |  | **C** |
| ***Abudefduf vaigiensis*** | **Indo-Pacific sergeant** |  | **C** |
| ***Amblyglyphidodon curacao*** | **staghorn damselfish** |  | **C** |
| ***Amblyglyphidodon orbicularis*** | **N/A** |  | **C** |
| ***Amphiprion barberi*** | **N/A** |  | **C** |
| ***Amphiprion chrysopterus*** | **orangefin anemonefish** |  | **C** |
| ***Chromis atripectoralis*** | **black-axil chromis** |  | **C** |
| ***Chromis atripes*** | **dark-fin chromis** |  | **C** |
| *Chromis iomelas* | half-and-half chromis |  | S |
| ***Chrysiptera parasema*** | **goldtail demoiselle** |  | **C** |
| *Chrysiptera talboti* | **Talbot's demoiselle** |  | S |
| ***Chrysiptera taupou*** | **southseas devil** |  | **C** |
| ***Dascyllus aruanus*** | **whitetail dascyllus** |  | **C** |
| ***Dascyllus reticulatus*** | **reticulate dascyllus** |  | **C** |
| ***Dascyllus trimaculatus*** | **threespot dascyllus** |  | **C** |
| ***Plectroglyphidodon dickii*** | **blackbar devil** |  | **C** |
| ***Pomacentrus auriventris*** | **goldbelly damsel** |  | **C** |
| ***Pomacentrus imitator*** | **imitator damsel** |  | **C** |
| *Pomacentrus maafu* | N/A |  | S |
| ***Pomacentrus tripunctatus*** | **threespot damsel** |  | **C** |
| ***Pomacentrus vaiuli*** | **ocellate damselfish** |  | **C** |
| ***Pomacentrus wardi*** | **Ward's damsel** |  | **C** |
| ***Stegastes albifasciatus*** | **whitebar gregory** |  | **C** |
| **Pseudochromidae** | | | |
| ***Pseudochromis fuscus*** | **brown dottyback** |  | **C** |
| **Scaridae** | | | |
| *Bulbometopon muricatum* | bumphead parrotfish | kalia | I |
| ***Calotomus spinidens*** | **spinytooth parrotfish** |  | **C** |
| *Chlorurus microrhinos* | steephead parrotfish | ulurua | I |
| ***Scarus frenatus*** | **bridled parrotfish** |  | **C** |
| ***Scarus niger*** | **dusky parrotfish** |  | **C** |
| **Scombridae** | | | |
| *Acanthocybium solandri* | wahoo | wahoo | **I** |
| **Serranidae** | | | |
| *Cephalopholis argus* | peacock hind | kawakawaloa | I |
| *Cephalopholis miniata* | coral hind | timo | I |
| ***Cephalopholis urodeta*** | **darkfin hind** | **kawakawadamu** | **C** |
| *Epinephelus fuscoguttatus* | brown-marbled emperor | delabulewa | I |
| ***Epinephelus merra*** | **honeycomb grouper** | **seni kawakawa** | **C** |
| *Epinephelus polyphekadion* | camouflage grouper | kawakawa | I |
| *Plectropomus leopardus* | leopard coralgrouper, red salmon cod | donu | I |
| **Siganidae** | | | |
| *Siganus argenteus* | forktail rabbitfish | mulu | I |
| ***Siganus spinus*** | **scribbled rabbitfish** | **nuqanuqa** | **C** |
| ***Siganus vermiculatus*** | **vermiculated spinefoot** | **nuqa/gusula** | **C, I** |
| **Sphyraenidae** | | | |
| *Sphyraena barracuda* | great barracuda | ogo, ogolevu | I |
| **Terapontidae** | | | |
| ***Mesopristes kneri*** | **orange-spotted therapon** |  | **C** |
| *Terapon jarbua* | crescent-banded grunter | qeawa | I |
| **Zanclidae** | | | |
| *Zanclus cornutus* | moorish idol | latinidaveta | I |

1. Fishermen say that about eight species of *labe* exist in Nagigi, but it is uncertain which of the many species of Labrids recorded in the area would be considered *labe.* [↑](#footnote-ref-1)
